# Host and Viral Zinc-Finger Proteins in COVID-19

**DOI:** 10.3390/ijms23073711

**Published:** 2022-03-28

**Authors:** Sabrina Esposito, Gianluca D’Abrosca, Anna Antolak, Paolo Vincenzo Pedone, Carla Isernia, Gaetano Malgieri

**Affiliations:** Department of Environmental, Biological and Pharmaceutical Sciences and Technologies, Università della Campania, Luigi Vanvitelli, Via Vivaldi 43, 81100 Caserta, Italy; sabrina.esposito@unicampania.it (S.E.); gianluca.dabrosca@unicampania.it (G.D.); anna.antolak@unicampania.it (A.A.); paolovincenzo.pedone@unicampania.it (P.V.P.); carla.isernia@unicampania.it (C.I.)

**Keywords:** zinc finger, SARS-CoV-2, coronavirus, COVID-19, therapy

## Abstract

An unprecedented effort to tackle the ongoing COVID-19 pandemic has characterized the activity of the global scientific community over the last two years. Hundreds of published studies have focused on the comprehension of the immune response to the virus and on the definition of the functional role of SARS-CoV-2 proteins. Proteins containing zinc fingers, both belonging to SARS-CoV-2 or to the host, play critical roles in COVID-19 participating in antiviral defenses and regulation of viral life cycle. Differentially expressed zinc finger proteins and their distinct activities could thus be important in determining the severity of the disease and represent important targets for drug development. Therefore, we here review the mechanisms of action of host and viral zinc finger proteins in COVID-19 as a contribution to the comprehension of the disease and also highlight strategies for therapeutic developments.

## 1. Introduction

SARS-CoV-2 (Severe Acute Respiratory Syndrome CoronaVirus 2) is the new type of coronavirus responsible for the pandemic spread of the severe and highly infectious disease called COVID-19 (COrona VIrus Disease 2019) [1]. This positive single-stranded RNA virus belongs to the *Coronaviridae* family (genus *Betacoronavirus*), just like the previous Middle East Respiratory Syndrome (MERS-CoV) and SARS-CoV-1 [2,3]. It was first found to infect humans in the Chinese city of Wuhan at the end of 2019 and, because of its enormous speed of diffusion among human population, it has become in a few months a worldwide emergency (WHO declared COVID-19 a pandemic on 11 March 2020) with devastating effects in terms of infections and deaths as well as in terms of socio-economic impacts [4].

While the mortality rate is debated and it is quite different in the diverse countries, varying from 0.5 to 7.7% [5,6,7], a few factors have emerged to be associated with the severity of the disease such as age, sex (males appear to be more susceptible to severe forms), pre-existing chronic metabolic diseases including diabetes, cardiovascular disease, and obesity [8]. SARS-CoV-2 infects the human host penetrating the lung epithelial cells: the virus spike (S) glycoprotein associates with the cellular receptor ACE2 (Angiotensin I Converting Enzyme 2) and the inoculation of the virus RNA into the host cytoplasm follows [4,9].

At this point, the virus uses viral proteins in cooperation with the host apparatus to replicate the viral genome and assemble new viruses that are released to infect new individuals [3]. Full-length negative-sense RNA copies are produced at the beginning of the infection that act as further templates for synthesizing positive-sense genomic RNA [10].

SARS-CoV-2 RNA genome is highly similar to SARS-CoV-1, is approximately 30 kb long, contains a 5′ cap and a 3′ poly A tail [11,12], and the study of its sequence allowed the closest relative of this pandemic virus in horseshoe bats to be found [4].

Overall, it encodes about thirty proteins: four structural proteins, Spike (S), Nucleocapsid (N), Envelope (E), and Membrane (M), involved in forming the viral envelope and in the packaging of the RNA genome; the other proteins cover a range of functions during the life cycle of SARS-CoV-2. Among the latter, sixteen non-structural proteins (labelled as nsp1–16) [13,14] take part in replication and transcription of the viral genome. While intracellular antiviral defences can inhibit the replication of SARS-CoV2 and reduce COVID-19 severity, which is also influenced by inherited genetic factors; in many cases infection can lead to endothelial cell dysfunction, systemic inflammation, and coagulation/thrombosis [15,16]. The innate immune reaction leading to pro-inflammatory cytokines and interferon synthesis is the first line of defence to control viral replication. SARS-CoV-2 infection can, however, culminate in a cytokine storm that prompts inflammation, local tissue harm, and systemic multi-organ failure [17].

COVID-19 is recognized as a cytokine release syndrome and endothelial disease [18] since the disruption of the endothelial homeostasis follows the cytokine storm with vascular endothelium that becomes malfunctional.

**Figure 1:**
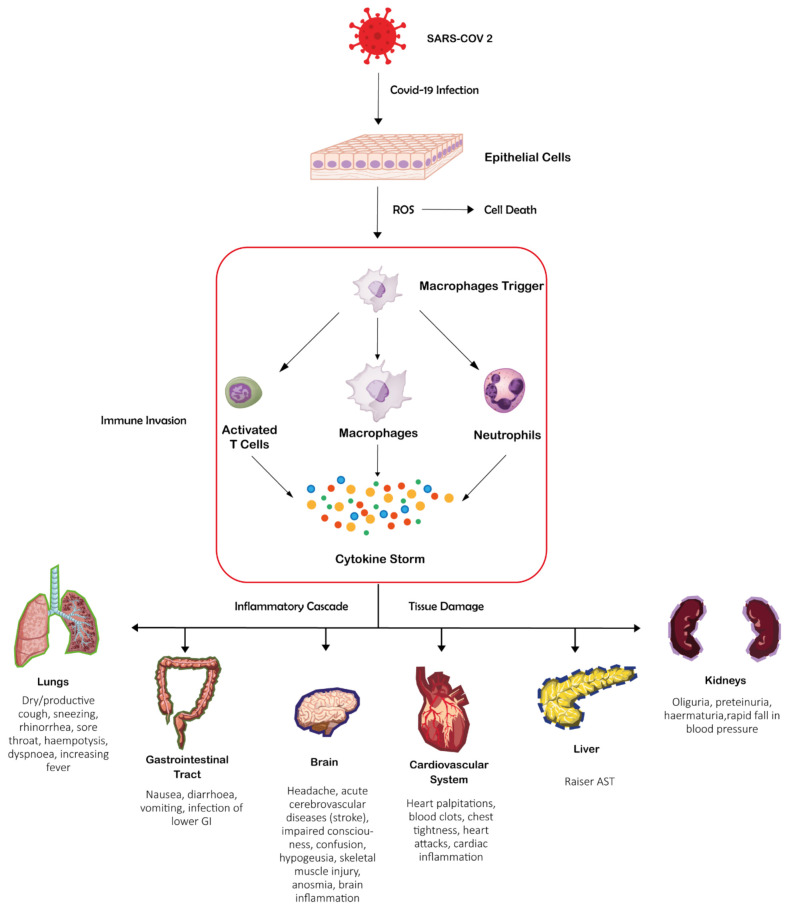
SARS-CoV-2 infection can induce a cytokine storm that prompts inflammation, local tissue harm, and systemic multi-organ failure.

Severity of COVID-19 [19] is correlated to the unique inflammatory profile of patients with high serum levels of multiple cytokines and chemokines/growth factors [20]: TNF-α, IL-1, 6 and 12, IFN-gamma, and TGF-beta, CCL2, CXCL9 and 10, and IL-8. Such an inflammatory profile causes several aspects of endothelial dysfunction [21], including hyper-inflammation, coagulation [22,23], and thrombosis [24] (Figure 1). This inflammatory profile in turn leads to acute respiratory distress, failure of microcirculation, and multi-organ dysfunction that can culminate with the patient’s death [7,25,26]. Specific treatments are on their way, and the study of molecular details of SARS-CoV-2 functional components, of viral-host protein interactions, and of antiviral response could provide essential objectives for drug development.

In this context, the urge to write this review came from the observation that proteins containing ZNF (Zinc-Finger), both belonging to SARS-CoV-2 or to the host, play critical roles in COVID-19 participating in regulation of viral replication and life cycle, interaction with host and negative regulation of virus release, interferon response, antiviral response, and myeloid cell differentiation.

Differentially expressed ZNF proteins and distinct activities could be important in determining the severity of the disease and the survival outcome of patients [27,28,29]. ZNF protein expression and activity of patients is noticeably different following SARS-CoV-2 infection [29]. Lung epithelial cells show a significantly decreased ZNF protein activity in mild and severe patients when compared with healthy people. Furthermore, ZNF proteins activity of some immune cells, such as macrophages, CD8 T cells, CD4 T cells, and NK cells increases in mild patients, but significantly reduces in severe patients [27,28,29].

ZNF family proteins can inhibit SARS-CoV-2 and increase host cellular defence through transcriptional regulation and act as antiviral effector proteins. ZNF transcription factors can activate type I interferon signalling and several antiviral genes [27,28,30,31], promote immune cells activation and maturation [32,33], but also directly or indirectly upturn the content of antiviral antibodies [33,34].

Therefore, as a comprehensive and unifying study of the mechanisms of action of ZNF proteins could be important in the development of anti-COVID-19 therapies, our purpose is to focus, among the many biological activities, on the roles of ZNF proteins in COVID-19 viral infection by examining the reported results.

## 2. Zinc Fingers

ZNFs are chemically and functionally unique protein domains characterized by the complexation of a Zn(II) ion by a different combination of Cys and His. The metal coordination guarantees the stability of high organized structures [35,36,37]. Although there are several cases of ZNF where the metal coordination sphere is also made by Asp and Glu residues, Cys and His are highly preferred to bind the zinc with a tetrahedral geometry that guarantees the correct spatial orientation of the domain chain [38,39]. The metal-bound protein folding is supported by the presence of hydrophobic residues whose role is not only to stabilize the correct structure with the formation of a hydrophobic core, but also to promote inter-domain interaction with other ZNF [40,41]. All these forces, together with side chain–side chain or main chain–side chain electrostatic interactions and formation of hydrogen bonds are crucial for the thermodynamic stability of the entire domain [42,43]. The differences within this broad class of protein domain are linked to the consensus amino acids sequence that leads to the classification of the ZNF peptides on the basis of their structural peculiarity.

**Figure 2:**
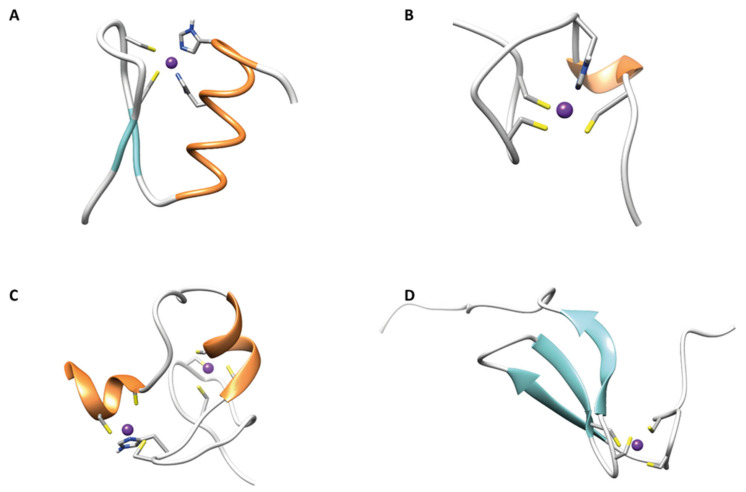
(**A**) First ZNF of TFIIIA (*ββα* zinc finger) (PDB code 1TF3); (**B**) C-terminal ZNF of HIVNCp7 (gag knuckle) (PDB code 2L44); (**C**) C-terminal ZNF of RING finger protein 141 (treble clef finger) (PDB code 5XEK); (**D**) N-terminal ZNF of TFIIB (zinc ribbon) (PDB code 1PFT).

In Figure 2 the ribbon drawings of different classes of ZNF are reported.

### 2.1. ββα Zinc Fingers

This family of ZNF is the most abundant in Eukaryotes, being encoded by more than 3% of human genes [44]. This class of peptides is involved in several cellular processes, and they can be found in proteins as a single functional domain, coupled with other zinc fingers or associated with other independent domains [45,46,47]. The consensus sequence (Y/F)-X-C-X_2,4_-C-X_3_-X-L-X_2_-H-X_3,4_-H consists of ~30 amino acids with a CCHH coordination sphere and a three residue hydrophobic core. Recently, larger *βββαα* zinc-binding globular domains, involved in DNA-binding and genes regulation, were also found in Prokaryotes [48,49,50].

### 2.2. Gag Knuckle

The gag knuckle ZNFs have shorter structure (20 AA) represented by two truncated *β*-strands linked by a zinc knuckle followed by a loop or a short helix. In this group the Zn(II) is coordinated by a CCHC motif [51], as found in many viral proteins belonging to this class [51,52].

### 2.3. Zinc Ribbons

Zinc ribbons are composed by two Zn(II)-biding sub-sites named zinc knuckles made by two different *β*-hairpins located at the N- and C-terminal of the structural fold. The C-terminal one can form H-bonds with another *β*-strand of the zinc ribbon fold [51,53]. Some members of this class have hydrophobic residues on the *β*-sheet that are responsible of hydrophobic interactions with proteins adopting the same fold [54].

### 2.4. Additional Zinc Finger-Like Motifs

A lot of poorly described protein structures are thought to be stabilized by the coordination of a zinc ion, existing as independent domains. They are grouped together on the basis that they do not form regular secondary structure elements upon the coordination of Zn(II). Most of the proteins belonging to this group are transcription factors and are listed as CCCH- and CCHHC-type fingers [51].

### 2.5. Treble Clef Fingers

A huge number of structural Zn(II) binding proteins, even with different cellular functions, are known as treble clef ZNFs. They share the same structural features, consisting of an *α*-helix at the C-terminus and a zinc knuckle with a CXXC sequence *β*-hairpin at the N-terminus. RING, LIM, FYVE, and PHD ZNF domains belong to this group and are often found coupled in tandem in multidomain proteins [55].

## 3. Roles of Host ZNF Proteins in COVID-19

### 3.1. Gender-Related Prognosis: The Role of ZNF Proteins

Men and women appear to have a substantial difference in mortality and severe illness rates related to COVID-19 [56,57,58]. Global data that analyse the infection and mortality distribution in COVID-19 patients of different genders suggest that survival outcome is superior in female patients compared to male [57,59,60]. Also keeping in consideration that men show an infection rate of 1.58% higher than women, their survival probability appears lower [57,59,60,61].

The molecular mechanisms that induce different prognostic outcomes between male and female patients has been recently studied by analysing mRNA expression levels, proteins activities, and clinical indicators [29].

The study has revealed that SARS-CoV-2 infection leads to a significant upregulation of two master regulators such as STAT1/STAT2 and ZNF proteins. In particular, the latter appear to play critical anti-SARS-CoV-2 roles as their function is in strong negative correlation to the virus load while shows a positive correlation with certain immune cells.

The infection promotes the expression of several ZNF proteins (e.g., ISG, Interferon Stimulated Genes) to directly neutralize SARS-CoV-2 [62,63,64,65]. Furthermore, these proteins can also function as transcription factors to regulate target genes engaged in anti-SARS-CoV-2 battle. As a result, ZNF proteins show two key activities in COVID-19: transcription factor activity and antiviral activity [27,32,62,63,66,67,68].

Female patients in response to SARS-CoV-2 infection have exhibited the expression of more ZNF proteins and stronger transcriptional activities with respect to male patients [29]. They also show higher ratio and activity of lymphocytes [69] and fewer monocytes than male patients, indicating that ZNF proteins and immune cells can reciprocally stimulate in response to SARS-CoV-2 infection [33].

The gender-related variation of ZNF proteins activities of several lymphocytes cell populations have prognostic effects in COVID-19.

### 3.2. ZAP Host Zinc Finger Inhibits SARS-CoV-2 Replication

The host innate immune system includes antiviral restriction factor proteins whose function is to limit the variety and severity of virus infections [70].

ZAP (zinc finger antiviral protein), produced by animal cells including humans, is a broad-spectrum antiviral restriction protein known to inhibit replication of several RNA viruses and pathogens, including influenza A virus, alphaviruses, hepatitis B virus, filoviruses, and retroviruses [62,71,72,73,74,75,76,77]. In addition, it is a key regulator of integrated viral components and their activity inside the human genome [78].

**Figure 3:**
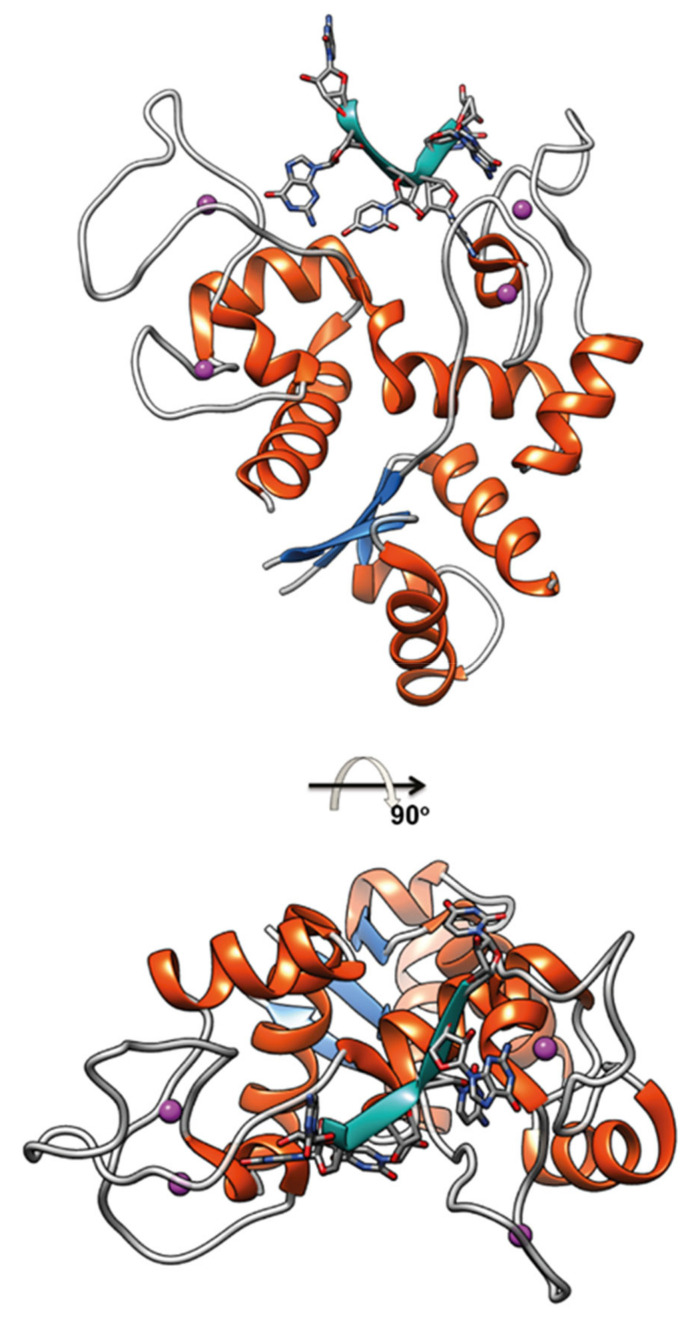
ZAP N-terminal domain binding a CG-rich single strand of RNA; CG dinucleotide is the key element targeted by ZAP (PDB code 6L1W).

ZAP is a Cys_3_His-zinc finger protein, interferon-inducible, that in the course of infections can distinguish and induce the degradation of viral mRNAs and proteins, exerting its antiviral action by activating T cells. ZAP N-terminal domain contains four Cys_3_His-ZNFs that constitute the RNA binding interface that binds CpG-rich sequences in viral RNA (Figure 3), little represented in the host transcriptome [79,80]. Such binding suppresses virus replication by interfering with the assembly of the initiation complex for translation of the target virus mRNA [81]. In addition, ZAP engages several RNA processing proteins to degrade target RNAs [76,82,83,84].

Although SARS-CoV-2 shows a somewhat low frequency of CpG dinucleotides that could have facilitated its spread, endogenous ZAP appears as a significant effector of the antiviral innate response against this pandemic pathogen infection, reducing the levels of expression of the viral RNA [85]. Nchioua et. al. [66] have demonstrated that endogenous ZAP expression significantly contributes to restriction of the virus replication in human lung cells upon treatment with IFN. They have proved that the cells commonly used in SARS-CoV-2 research [86,87], the human epithelial lung cancer cell lines Calu-3 and A549 and primary human lung fibroblast constitutively express ZAP. Treatment with interferons type I (α/β), II (γ), and III (λ) all strongly inhibit SARS-CoV-2 and enhanced the expression of ZAP. Particularly, the type II (γ) is the most potent in inducing ZAP expression in human lung cells (up to 8-fold) and in reducing SARS-CoV-2 replication.

This documented capability of ZAP to restrict SARS-CoV-2 pandemic viral pathogen might encourage the development of therapies against COVID-19 and IFN-γ is currently considered for treatment of COVID-19 patients [88,89].

### 3.3. ZNFX1 in COVID-19

Qin et al. [29] have reported that upon Sars-CoV-2 infection the activity of ZNFX1 (zinc finger NFX1-type containing 1) negatively correlated to SARS-CoV-2 load. ZNFX1 is a ZNF protein belonging to the helicase super family 1 whose antiviral activity has been first reported by Wang et. al. in 2019 [32]. ZNFX1 is a mitochondrial-localised sensor for RNA viruses that induce IFN production and IFN-stimulated gene expression in the early stage of RNA virus infection [32,66,67,90]. Upon virus infection, ZNFX1 immediately interacts with mitochondrial antiviral signalling protein to initiate the type I IFN antiviral responses.

### 3.4. Role of Host Zinc Finger CCHC-Type Containing 3 (ZCCHC3)

An interactome analysis [91] conducted by means of affinity purification and mass spectrometry profiled 160 cellular proteins as interaction partners of the Nucleocapsid protein (N) of SARS-CoV-2 in HEK293T and/or Calu-3 cells. Among them, six proteins are associated to defence responses against viral infections. In particular, the zinc-finger protein ZCCHC3 was recognized as interaction partners of the SARS-CoV-2 N protein.

ZCCHC3 is a Cys_2_HisCys-protein that binds RNA and enables the recognition of intracellular RNA viruses activating anti-viral response mediated by IFNα-induced signalling and upregulating antiviral proteins (such as RNase L and PKR), known to degrade viral RNA and prevent its translation [92,93,94,95,96,97].

### 3.5. KLF2 Host Zinc Finger Protects against COVID-19 Associated Endothelial Dysfunction

KLF2 (Krüppel-like factor 2) belongs to the Krüppel-like family of ZNF transcription factors and bears a Cys_2_His_2_ ZNF-containing DNA binding domain. KLF2 is a key regulator of activation, differentiation, and migration processes in numerous cell types, including endothelial cells function and vascular homeostasis [98].

KLF2 plays a significant role in COVID-19 as SARS-CoV-2 significantly down-regulates KLF2 expression: COVID-19 patients have elevated serum levels of TNF-α and IL-1 and decreased KLF2 gene expression [99]. KLF2 downregulation induces monocyte adhesion, endothelialitis, and lympho-monocytic cells infiltration in these patients. For these reasons KLF2 is emerging as an important factor in COVID-19-induced endothelial dysfunction and vascular disease and thus as a promising target for therapeutic intervention.

Xu and co-workers [99] studied the mechanism of endothelial dysfunction caused by SARS-CoV-2 infection by treating human endothelial cells HUVEC with serum from COVID-19 patients and by analysing gene (by qPCR and RNA-sequencing data) and protein expression.

In treated endothelial cells, a downregulation of the expression of KLF2 by components of the cytokine storm TNF-α and IL-1 is observed as well as an increase of monocyte adhesion due to elevated ICAM1 and VCAM1 protein expression (markers of endothelial inflammation).

The role of KLF2 in the endothelial dysfunction has been further underlined by the treatment of endothelial cells with KLF2 siRNA (siRNA against KLF2). Such treatment reduced KLF2 gene expression resulting in a depletion of this protein that further enhanced monocyte adhesion to endothelial cell due to the treatment with the serum from COVID-19 patients. On the other hand, genetic or pharmacological activation of KLF2 reversed multiple aspects of the induced endothelial dysfunction thus proposing KLF2 activation as a plausible strategy to improve COVID-19 associated endothelial dysfunction [99].

### 3.6. MADP1 and SARS-CoV-2 RNA Synthesis

Coronaviruses RNA replication takes place in the host cytoplasm and is controlled by host cell proteins. Once in the host cell, the virus unleashes its 5′-capped genome that is a single-stranded positive-sense RNA similar to host mRNA [100].

mRNA 5′ untranslated regions (UTRs) provide the ribosome entry point and can assume complicated secondary and tertiary structures which can regulate translation initiation. The 5′ UTRs interact with host factors and such interaction plays important roles in the replication, disease evolution, and aetiology [101,102,103]. In particular, during the intermittent replication process of Sars-CoV-1 genome, SL1 (RNA stem-loop 1), and SL2 support viral replication and transcription by interacting with the host MADP1 aided by nsp1 protein [103,104].

MADP1 encompasses two conserved RNA-binding domains, the RNA recognition motif 1 and a Cys_2_HisCys ZNF [104] and has a critical role in determining the replication efficiency within host cells [103].

Variations in UTRs may affect the activity of viral RNA folding and packaging in some viral genomes [105]. SARS-CoV-2 genome is about 29–31 kb and its 5′ UTR presents various stem loops named SL1–3, SL4A, SL4B, and SL5 [106]. One of the most frequent 5′ UTR variants in the SARS-CoV-2 genome is the C241T emerged in March 2020 [107]. Such mutation has an undefined significance [108], however its effects have been studied in-silico [104]. The study shows how C241T mutation changes the folding of SL4 RNA decreasing the stability of 5′ UTR and affecting its interaction with host transcription factor MADP1. The resulting weaker interaction of the host MADP1 with this mutant SL suggests a reduced efficiency of virus replication.

### 3.7. The Zinc Finger DHHC Domain-Containing (ZDHHC) Palmitoyl Transferase Proteins in Viral Infection

SARS-CoV-2 infection starts with the interaction of the virus S protein and the host receptor ACE2 [109]. In order to accomplish its task, the S protein experiences complex posttranslational modifications such as glycosylation [110,111,112], phosphorylation [113], and palmitoylation.

Palmitoylation is the covalent attachment addition of palmitic acid, a 16-carbon saturated fatty acid to cysteine thiol group of proteins (S-palmitoylation) by the zinc finger Asp-His-His-Cys (DHHC) domain-containing (ZDHHC) palmitoyl acyltransferase (PAT) family proteins [114].

The function of such modification is substrate-specific [115] as it depends on the specific protein considered: it enhances proteins hydrophobicity enabling their membrane association and plays a major role in their subcellular trafficking through membrane compartments [114], and in modulating protein–protein interactions. ZDHHC proteins are encoded in humans by 23 distinct genes [115]. All of them are predicted to cross the membrane multiple times with four to six transmembrane domains while their catalytic DHHC domain is on a cytosolic loop. Most ZDHHC enzymes are associated with the endoplasmic reticulum and Golgi apparatus while a minor number with the plasma membrane and endosomes [114].

The palmitoylation of SARS-CoV-2 S has been proven necessary for SARS-CoV-2 entry and viral infectivity [116,117] since it is critical for S-mediated cell fusion and syncytia formation [118]. S protein palmitoylation occurs at multiple sites, particularly the N-terminal Cys15 and nine cysteines at C-term cys-rich cytoplasmic tail [118] and is promoted by multiple ZDHHCs: ZDHHC2, ZDHHC3, ZDHHC4, ZDHHC5, ZDHHC8, ZDHHC9, ZDHHC11, ZDHHC14, ZDHHC16, ZDHHC19, and ZDHHC20. It is not yet clear whether different ZDHHCs are redundant, palmitoylate different sites in S or are located in different cellular compartment performing their functions in different intracellular locations.

Table 1 summarizes the human host ZNF proteins involved in COVID-19.

## 4. Roles of Viral ZNF Proteins

Almost all viruses have developed molecular tools to evade cellular immune response.

Coronaviruses have long single stranded RNA genome that are particularly sensitive to the antiviral innate response of the host cells.

For this reason, Coronavirus replication is an extremely coordinated process that implicates an array of non-structural replicase proteins (nsp) that not only regulate the transcription and replication of the genome but also interfere with the antiviral response mechanisms of the host cells.

SARS-CoV-2 genome bears different reading frames and its replicase unit involves 16 nsps (nsp 1–16), encoded by the two open reading frames ORF1a and ORF1b that are translated through a ribosomal frameshift mechanism [13,14].

This process supports the synthesis of PP1a and PP1ab, two large precursor polyproteins that are supplementary processed into distinct polypeptides by PLpro (the papain-like protease) and 3CLpro (3C-like protease), two cysteine proteases encoded inside PP1a itself.

PP1a and PP1ab are cleaved by these proteases at multiple sites allowing the 16 nsps release and maturation. Nsps are cooperatively engaged in the assembly of a membrane associated Replication-Transcription Complex (RTC) [119] directly responsible to reproduce the viral genome and generate mRNA. To fulfil this task, some nsps (nsp3, 4, and 6) hold transmembrane domains which mediate RTC recruitment to a system of vesicles originating from the endoplasmic reticulum [120]. How the different proteins composing the RTC are arranged to form the complete complex remains unclear. A recent study [121] proposes a molecular model of the SARS-CoV-2 RTC according to which RTC superstructure is built around an nsp15 hexamer.

### 4.1. SARS-CoV-2 Papain-Like Protease (PLpro)

As mentioned, SARS-CoV-2, just as SARS-CoV-1, encodes four structural proteins involved in forming the viral envelope and in the packaging of the RNA genome and sixteen non-structural proteins (labelled as nsp1–16) [14,122] that take part in replication and transcription of the viral genome. The nsps are cleaved from PP1a and PP1ab by the 3C-like cysteine protease (3CLpro, also known as the main protease (Mpro) or non-structural protein 5 (nsp5)) and the papain-like protease (PLpro, the protease domain autocleaved from nsp3) [123,124,125,126].

PLpro is also essential for the RTC formation [127,128] and is involved in cleaving proteinaceous post-translational modifications on host proteins [129,130,131]. It contains a Cys_4_ ZNF that tetrahedrally coordinates a zinc ion belonging to the “zinc ribbon” fold group [51,132] and it is essential for catalysis because it maintains the structural integrity of PLpro [122,133,134,135,136,137].

It is commonly assumed [138] that PLpro has a protease role for which it cleaves three sites between nsp1/2, nsp2/3, and nsp3/4 [127] and a deubiquitinating and deISGylating role for which it deconjugates ubiquitin and interferon-stimulated gene product 15 (ISG15) from their substrate signalling proteins (e.g., IRF3, the interferon responsive factor 3) and attenuates type I interferon responses [139]. ISG15 is a ubiquitin like protein considerably upregulated in antiviral response. It can be conjugated to signalling proteins and therefore enhance antiviral signalling pathways [139,140]. PLpro has thus both peptidase and isopeptidase activities [141].

PLpro from SARS-CoV-2 shares 83% sequence identity with PLpro from SARS-CoV-1 [139] but shows some differences: the first preferentially cleaves ISG15 while the latter mainly targets ubiquitin chains.

Recent evidences from in vitro and in-cell studies have evidenced how nsp3 proteins from SARS-CoV-1, MERS-CoV, and NL63-CoV interfere with the E3 ubiquitin ligase RCHY1 (the RING finger and CHY zinc finger domain-containing protein 1) [142,143,144,145]. Given this high degree of identity, a similar role has been suggested for SARS-CoV-2.

RCHY1 protein, also known as Pirh-2, contains a N-terminal CHY zinc-finger domain and a C-terminal RING-finger domain [142], and performs an E3 ubiquitin ligase activity that promotes p53 degradation independently of Mdm2 [142].

PLpro domain interacts with RCHY1 stabilizing it and enhancing the degradation of endogenous p53 [146]. In this task, PLpro acts synergistically with the SARS unique domain (SUD), a structural domain not present in other coronaviruses [142].

The genome guardian p53 known for its crucial role in responding to DNA damages plays a central part also in the innate host immune control of viral infections. It organises diverse signalling pathways originating from many different cellular receptors and sensors [147] playing a positive role in antiviral infection, inhibiting the replication of SARS-CoV-1 [142] and other viruses [147,148]. PLpro/RCHY1 interaction, promoting p53 degradation, results in inhibition of apoptosis and interferon innate immune signalling [149] that aids the coronavirus’s evasion of the host’s innate immune responses.

### 4.2. SARS-CoV-2 nsp14

SARS-CoV-2 nsp14 is a 60 kDa protein, highly conserved within the Coronaviridae family [150], that shares with its homologous protein from SARS-CoV more than 95% amino acid sequence identity [151,152].

Nsp14 major functions include an exoribonuclease activity and methyl transferase. It is also involved in other key functions such as recombination of the virus genome and control of the immune system [153]. Its N-terminus bears a 3′-to-5′ exoribonuclease (ExoN) while the C-terminus holds the N7-methyltransferase (N7-MTase) domain [154,155,156].

The ExoN catalyzes nucleoside monophosphate removal from nucleic acids in the 3′-to-5′ direction, essential for the virus replication proofreading activity, as it is proposed to be responsible for the removal of mismatched nucleotides from the 3ʹ end of the growing RNA chain [157,158]. This feature that improves the fidelity of replication is absent in other RNA viruses. ExoN is a distant member of the DEDD superfamily that contains four conserved residues (aspartic acid, glutamic acid, aspartic acid, aspartic acid) that form two metal-binding sites fundamental for the DNA and RNA exonuclease activity [155,156]. Substantial differences have been outlined between nsp14 ExoN and the members of this superfamily [150,156]. While Nsp14 ExoN alone shows exoribonuclease activity with a similar two-metal-ion–assisted mechanism for removal of mismatched nucleotides, a 35-fold increase in this activity is found when nsp14 is bound to nsp10, pointing out a role for nsp10 in the stabilization of the ExoN active site [150,159,160]. The active site also differs from that of the DEDD superfamily as in nsp14 one of the aspartates is substituted by a glutamate and a histidine residue completes the site, thus obtaining an Asp-Glu-Glu-His-Asp catalytic center [150]. Another notable difference is the presence of two zinc fingers essential for ExoN activity: the first ZNF, a Cys_3_His motif, has mostly a structural role as its mutation results in insoluble nsp14 [161]; the second ZNF, a HisCysHisCys, is suggested to have a role in catalysis as it is close to the catalytic core and its disruption through mutations abolished the enzymatic activity [150]. The first ZNF is close to the interaction site with nsp10 and it is suggested that it might affect the interaction nsp14/nsp10, thus influencing ExoN catalytic activity.

The N7-MTase domain, involved in mRNA capping and host immune response evasion [162,163], includes nsp14 third ZNF formed by three cysteines and one histidine. This ZNF is located at the C terminus of the N7-MTase and bears an additional twelve residues α-helix that stabilizes the local hydrophobic environment. Truncation of this region has been shown to strongly weaken or abolish the nsp14 methyltransferase activity [164].

### 4.3. SARS-CoV-2 nsp10

Nsp10 is a ~140 amino acid protein whose alignment with SARS-CoV-1 provides more than 97.1% of sequence identity [152]. It can be found exclusively in viruses and has a crucial role in RNA replication. It is a ZNF protein of the LIM type that bears two ZNFs, the first coordinates a zinc ion by means of three-cysteine and one-histidine side chains while the second ZNF metal site is composed by the side chains of four cysteines and can be classified as a “gag-knuckle” motif [51]. These zinc coordinating residues are highly conserved across Betacoronaviruses underlining the importance of this metal ion coordination. As reported above, it interacts with nsp14, acting both as a stimulatory and a scaffolding protein. Nsp10 also has the same stimulatory role for nsp16 activity, which is a 2′-O-methyltransferase [165]. The modification induced by nsp16 precludes the recognition and degradation of viral RNA by the host innate immune system [166,167].

### 4.4. SARS-CoV-2 nsp13

Nsp13 is a 67 kDa protein that shows a five-domain architecture. It is a member of the helicase superfamily 1B and it catalyzes, with a 5′ > 3′ polarity, the unwinding of double-stranded DNA or RNA, utilizing the energy of nucleotide triphosphate hydrolysis [168,169,170]. Sars-COV-2 nsp13 is the most conserved nsp as it differs from its homolog from Sars-COV-1 for only one amino acid (V570I). Its five-domain architecture assembles together a N-terminal zinc binding, a helical “stalk”, a beta-barrel 1B, and 2 tandem C-terminal RecA-like helicase domains. The “RecA like” domains are involved in nucleotide binding and hydrolysis. The zinc binding domain coordinates two structural zinc ions via a RING like module and an additional zinc ion via a treble-clef ZNF [169]. The first zinc ion is coordinated by four cysteine side chains, the second by a Cys_2_His_2_ coordination, and the third by a Cys_3_His [169]. The zinc-binding domain serves as an interface with other components of the replicative machinery.

Two copies of Nsp13 interact with nsp7, nsp8, and nsp12 within the multi-subunit RNA dependent RNA polymerase [171]. This interaction considerably stimulates nsp13 helicase activity. Within this complex, Nsp13 zinc-binding domains interact with Nsp8 and nsp12 [171,172,173,174]. Besides its helicase activity, the nsp13 active site also has RNA 5′ tri-phosphatase activity [175], thus indicating an additional crucial role for this protein in the formation of the 5′ mRNA cap.

### 4.5. SARS-CoV-2 nsp2

SARS-CoV-2 nsp2 localizes to endosomes and RTC and it has been connected to a number of viral processes; however, its structure and precise functions remain still need to be unveiled [176,177,178]. Though it is found also in SARS-CoV-1, MERS and in strictly related Coronaviruses, nsp2 shows significant sequence variations in the different species [178] probably due to mechanisms of adaptation under host-specific selection pressure. The N-terminal part of the protein appears to be slightly more conserved: the overall identity of SARS-CoV-2 nsp2 is 68% with its counterpart in SARS-CoV-1 and only 20% with MERS’s.

A number of host proteins are indicated as interacting with nsp2 by means of interactomes studies conducted on SARS-CoV-1, SARS-CoV-2, and MERS [176,179,180,181] suggesting an involvement of this protein in endosomal transport, translation repression, actin filament binding, and ribosome biogenesis. SARS-CoV-1 nsp2 can also interact with nsp7, nsp8, and other viral nsp [180,182] involved in viral replication.

The specific timing for nsp2 expression has been shown to have a key role in SARS-CoV-1 as its deletion leads to viable viruses with a deficiency in replication [183] and nsp2 expression from a different site in the genome does not recover this deficiency [184].

A recent study reported the structural characterization of the N-terminal of SARS-CoV-2 nsp2 [185] that has shown how it contains three zinc fingers, belonging to the Cys_2_His_2_, Cys_4_, and Cys_2_HisCys types. However, they also demonstrate that these domains do not play important roles in binding nucleic acids suggesting for these three ZNF different functions that remain to be unveiled.

### 4.6. SARS-CoV-2 nsp12

Sequence alignment of SARS CoV-1 and SARS CoV-2 nsp12 gives over 96% sequence identity and above 99% sequence similarity [152].

Nsp12 is the RNA-dependent RNA polymerase (RdRp) that in complex with a nsp7/nsp8 heterodimer and a monomer of nsp8 makes copies of viral RNA. Nsp12 alone exhibits poor activity in RNA synthesis: the formation of the complex lowers its dissociation rate to RNA [152,186]. Nsp12 is 932 amino acids long that includes a *β*-hairpin, an extended NiRAN domain (nidovirus RdRp-associated nucleotidyltransferase domain), an interface domain and a C-terminal RdRp domain [187].

SARS-CoVs nsp12 contains two zinc-binding sites composed by residues that are highly conserved across the CoV family: the first is in the NiRAN domain and it is a HisCys_3_ motif; the second zin ion is coordinated by a CysHisCys_2_ sphere.

These metal-binding sites are far from known active or interaction sites (nsp12), suggesting for them the role of structural components of the nsp12 architecture instead of being directly involved in enzymatic activity. The fact that nsp12 binds structural zinc ions as seen for nsp3, 10, 13, and 14 underlines an extensive use of this metal ion for folding proteins of the RTC.

Table 2 summarizes the viral ZNF proteins involved in COVID-19.

## 5. Road to Therapy

An unprecedented effort by the global scientific community has been undertaken to tackle the COVID-19 pandemic. However, most experts suggest that it is unlikely that COVID-19 is to be completely eradicated, at least in the short period. The emergence of the latest omicron variant on November 2021 [188] would seem to prove them right. Even after the protection of high-risk groups and of a large part of the population by means of vaccination, evidences seem to show that we will have to coexist with this virus for a long time. For this reason, it is extremely important to introduce drugs specific to COVID-19 along with vaccination. The study of the molecular mechanisms underlying the host immune response and the “life” cycle of the SARS-CoV-2 virus are thus fundamental for the development of strategies for disease intervention.

Such work can be divided into three broad categories: the comprehension of the immune response to the virus, the host proteins that Sars-CoV-2 uses in its pathogenesis, and the functional role of the SARS-CoV-2 proteins. ZNF proteins are involved in all of them.

The ZNF Antiviral Protein appears to have the capability, especially upon treatment with IFN-γ, to restrict SARS-CoV-2 in spite of its important CpG suppression [66]. Clearly ZAP is only a member of the factors involved in the innate immune response and SARS-CoV-2 is capable of blocking the ribosomal translation of such factors [189]. However, the evaluation of combination therapies that include IFN-γ can be a valuable road to keep in consideration towards an immune therapy for treatment of COVID-19 that prompts further studies to understand whether the other kinds of IFN use different effectors to restrict this pandemic virus. Such a strategy is supported by the fact that ZNFX1 and ZCCHC3 are proteins involved in the anti-viral response mediated by IFN-induced signaling.

The so called “cytokines storm” released from COVID-19 patients is responsible for the frequently observed ARDS (acute respiratory distress syndrome) characterized by pulmonary endothelial dysfunction and lung inflammation. Inflammation caused by Sars-CoV-2 downregulates KLF2 and for this reason pharmacologic and genetic approaches to boost KLF2 levels have been tested by Xu and coworkers [99]. KLF2 activation (by atorvastatin and tannic acid, a natural KLF2 activator) and KLF2 overexpression (by KLF2 adenovirus) have been proven to significantly reduce patient-serum-induced monocyte adhesion to endothelial cells and have therapeutic potential in limiting endothelial dysfunction [99]. They have shown that treatment of HUVEC cells with atorvastatin (statins are activators of KLF2 [190]) in the presence of patients’ serum induces the expression of protective (anti-inflammatory, anti-angiogenesis, and anti-thrombotic) genes. Moreover, KLF2 overexpression via an adenoviral vector reverses patient-serum-induced endothelial dysfunction and controls the expression of genes related to endothelial dysfunction with the upregulation of vascular homeostasis related genes (KLF2, NOS3, and THBD) and antioxidant genes (GCLM and NQO1) and reduction of the expression of pro-inflammatory (VCAM1, CCL2, and DKK1), vasoconstrictive (EDN1), and pro-angiogenic (ANGPT2) genes.

Among the proteins reviewed in this article, an interesting target for anti-virus drug development is the palmitoylation of the S protein, the key of SARS-CoV-2 association with the receptor ACE2. S palmitoylation, mediated by ZDHHC, appears to have an important role in syncytia formation and virus entry. Recent studies have focused on S palmitoylation and showed how C75 (α-methylene-β-butyrolactone), a fatty acid synthase inhibitor, and 2-BP, a ZDHHC inhibitor, reduced the palmitoylation of S and syncytia formation [118].

An important research road for COVID-19 therapies is the development of antiviral agents targeting the RNA dependent RNA polymerase (RdRp), the multi-protein machinery responsible for SARS-CoV-2 genome replication and transcription. As described above, nsp12 activity, that is quite little unless nsp12 is bound to its nsp7 and eight co-factors, is central to the catalytic core of this multi-protein complex. Its central role prompted the research for nucleotide analogues that has led to the approval of Remdesivir [191] and Molnupiravir [192], while others, such as Galidesevir [193] or Favipiravir [194], are at different phases of development.

Remdesivir interferes with the RdRp decreasing the viral RNA production [195]; it inserts into the growing RNA chain causing irreversible premature termination. Galidesevir also causes premature RNA chain termination during polymerization [196].

Molnupiravir has a different mechanism of action as it induces SARS-CoV-2 RNA mutagenesis and like Remdesivir, it is capable of escaping viral RNA proofreading [197]. Favipiravir also causes the selective inhibition of RdpR [194] and induces lethal RNA transversion mutations stopping the viral RNA replication [198,199]. Another target for therapeutic interventions tackling RNA transcription and translation is nsp13, the helicase that precedes the replicative machinery, or the complex formed by nsp14, nsp16, and nsp10. The highly conserved sequence of nsp13 in nidoviruses, with only one residue substitution with respect to its homologous in SARS-CoV-2, suggests, in principle, the possibility of a broad-spectrum therapeutics targeting this viral family [200]. However, development of drugs targeting helicases is quite challenging given the huge number of cellular helicases. Small molecule drugs against viral helicases have been developed for herpes simplex virus [201] and hepatitis C [202]. Drugs targeting nsp13 should be designed to block its interaction with nsp7/8/12 complex or its functions (ATPase or helicase) such as specific inhibitors that compete with ATP for binding at the ATP binding site. Among the patented inhibitors, SSYA10-001, a 1,2,4-triazole compound, is the best characterized and has been proven capable of inhibiting ATPase and helicase activities of SARS-CoV nsp13 in a noncompetitive mode compared to nucleic acid and ATP substrate [203,204]. In addition, RNA aptamers containing adenosine/guanine-rich sequences have been proved capable of inhibiting nsp13 unwinding activity [205].

RNA capping mediates polyprotein translation and is extremely important to enhance viral genome stability inside the host cell [206]. The complex formed by nsp10/nsp14 and nsp10/nsp16 are responsible for SARS-CoV-2 RNA capping and represent another attractive therapeutic target also in light of the fact that the nsp10 exists exclusively in viruses but not in prokaryotes or eukaryotes. The objective is to hinder nsp10 binding to nsp14 and nsp16, therefore suppressing the stimulation of 3′-to-5′ ExoN and 2′-O’-MTase functions; nsp10-derived peptides have been shown to inhibit in vitro 2′-O’-MTase activity [207] and in vivo replication and pathogenesis of Sars-CoV-1 [165]. More recently, an in-silico exploration of potential natural inhibitors of nsp10 suggested Vidarabine as a potential SARS-Cov-2 nsp10 inhibitor [208].

Finally, the targeting of post-translational processing mediated by the papain-like protease (PLpro) also is a promising avenue toward the obtainment of effective drugs. Nsp3, with its multi-faceted proteolytic activity, is in fact an important target in inhibitors research with YM155 (an anti-cancer drug candidate in clinical trials) [209,210,211], tashinones (two active compounds from Chinese herbal medicine Salvia miltiorrhiza) [212,213] and GRL0617 (a compound previously reported to inhibit SARS-CoV PLpro) [214] showing promising activities. YM155 is an imidazolium-derived inhibitor of the anti-apoptotic protein survivin investigated for treatment of various cancers. YM155 inhibitory mechanism targets three “hotspots” in PLpro: the binding active site blocking substrate entry, partially occupies the ISG15 binding site, and the zinc finger motif causing conformational changes that affect the enzyme activity. Rut et al. have shown how tetrapeptide-derived inhibitors have the potential to block PLpro thus inhibiting SARS-CoV-2 replication and host cell evasion mechanisms [215].

## 6. Conclusions

The quest for finding efficacious treatments for COVID19 will be addressed by fundamental researches into the molecular mechanisms at the bases of the host immune response and of the replication cycle of the SARS-CoV-2 virus. Many studies are identifying a large number of ZNF proteins involved in SARS-CoV-2 infection and the study of the mechanisms of action of ZNF proteins may be important in the development of anti-COVID-19 therapies. Currently, only a few antiviral treatments for SARS-CoV-2 are available. Considering the average time frame for drug development, it is clear how the recent advancements in the SARS-CoV-2 knowledge and treatments are built on the bases of previous work on SARS-CoV-1, MERS-CoV, and other CoVs. A lot remains to be understood including, for example, key information on the RTC and on viral-host protein interactions. Nevertheless, the information gained to date from numerous vaccination and clinical trials together with a countless number of drug discovery studies has already importantly addressed therapeutic development. The studies conducted on S and N proteins as antigenic targets have successfully allowed the production of vaccines and antibody-based therapeutics while the studies of the RTC have paved the way to the approval of nucleotide derived antiviral drugs, including drug repurposing. Further studies, also based on the studies presented here, will build on the information summarized here towards new developments in therapeutics that should ideally lead to not expensive specific drugs that target non-hospitalized patients with mild to moderate symptoms.

## Figures and Tables

**Table 1:** Human zinc finger proteins in COVID-19 viral infection.

Protein Name	Activity	Regulation	Function	Pathologic Implications
ZAP (zinc finger antiviral protein)	antiviral	↑	Degrade viral RNA	inhibits SARS-CoV-2 replication
ZNFX1 (zinc finger NFX1-type containing 1)	transcription factor	↑	Induces interferon-stimulated genes (ISGs) expression	(antiviral innate immunity) restricts the replication of RNA viruses
ZCCHC3 (zinc finger CCHC-type containing 3)	antiviral	?	Interact with SARS-CoV-2 N protein	active virus defence responses
KLF2 (kruppel-like factor 2)	transcription factor	↓	Protects against COVID-19 associated endothelial dysfunction	monocyte adhesion and endothelial inflammation
MADP1	transcription factor	?	Replication factor in SARS-CoV-2 RNA synthesis	C241T mutant RNA SARS-CoV-2 reduces virus replication efficiency
ZDHHC (zinc finger DHHC domain-containing)	S protein palmitoylation	?	Critical for S-mediated SARS-CoV-2 entry	essential for viral infectivity

↑ refers up-regulation, ↓ refers down-regulation upon SARS-CoV-2 infection and ? is ambiguous.

**Table 2:** SARS-CoV-2 zinc finger proteins in COVID-19 viral infection.

Proteins Name	Activity	Function	Pathologic Implications
PLpro (papain-like protease)	protease stabilizes RCHY1 (suggested)	cleaves the 16 nsp proteins cleaves ISG15 p53 degradation	essential for viral RNA transcription and replication; attenuates type I IFN response; viral evasion of the host immune responses
nsp14 N-terminal domain	3′-to-5′ exoribonuclease	RNA proofreading	essential for viral RNA replication
nsp14 C-terminal domain	N7-methyltransferase	mRNA capping	essential for viral RNA translation and viral evasion of the host immune responses
nsp10	interacts with nsp14	stabilizes and stimulates nsp14	essential for viral RNA replication; found exclusively in viruses
nsp13	helicase co-factor of nsp7, nsp8 and nsp12	nucleotide binding and hydrolysis	stimulates the viral replicative enzymes activity
nsp2	interacts with host proteins	impede host protein synthesis	disruption intracellular signalling pathways
nsp12	RNA-dependent RNA polymerase interacts with nsp7 and nsp8	viral RNA synthesis	essential for viral RNA replication and transcription
